# Effects of Selected Antioxidants on Electroretinography in Rodent Diabetic Retinopathy

**DOI:** 10.3390/antiox14010021

**Published:** 2024-12-27

**Authors:** Radosław Dutczak, Marita Pietrucha-Dutczak

**Affiliations:** Department of Physiology, Faculty of Medical Sciences in Katowice, Medical University of Silesia, Medyków 18, 40-752 Katowice, Poland; fzjkatpl@sum.pl

**Keywords:** electroretinography, full-field ERG, multifocal ERG, pattern ERG, diabetic retinopathy, ocular diseases, retina, retinal ganglion cells, antioxidants

## Abstract

Electroretinography (ERG) is a non-invasive technique for evaluating the retinal function in various ocular diseases. Its results are useful for diagnosing ocular disorders and assessing disease progression or treatment effectiveness. Since numerous studies are based on animal models, validating the ERG results from animals is pivotal. The first part of this paper presents basic information on the types of ERG tests used on rodents, and the second part describes the recorded functional changes in rodents’ retinas when various antioxidant treatments for diabetic retinopathy were used. Our study showed that among the tests for diabetic retinopathy diagnosis in rodents, full-field ERG is accurate and the most commonly used, and pattern ERG and the photopic negative response of the flash ERG tests are rarely chosen. Furthermore, antioxidants generally protect retinas from functional losses. Their beneficial influence is expressed in the preserved amplitudes of the a- and b-waves and the oscillatory potentials. However, prolonging the drug exposure showed that the antioxidants could delay the onset of adverse changes but did not stop them. Future studies should concentrate on how long-term antioxidant supplementation affects the retinal function.

## 1. Introduction

Electroretinography (ERG) is a non-invasive and useful technique for evaluating retinal function in various ocular disorders, such as glaucoma, retinitis pigmentosa and diabetic retinopathy (DR) [1,2,3,4,5,6,7,8,9]. It measures the electrical responses of different retinal cells, including photoreceptors, bipolar cells, amacrine cells and ganglion cells, and is used both in clinics and laboratories. The information on the retinal cell function provided by ERG is useful not only in the diagnosis of ocular diseases but also in the assessment of disease progression or the effectiveness of treatment. Different retinal cell types can be distinguished by changing the stimulus conditions and the test type, so the selection of the ERG test and the parameters to be used during the recording, such as the stimulus intensity, electrode type and length of the dark adaptation, are crucial for accurate diagnosis of ocular disorders. The International Society for Clinical Electrophysiology of Vision (ISCEV) published a free-access website worldwide standard clinical protocols for electrophysiological examination [10]. However, all of these protocols were developed for humans and often require modification for use in animal models, which are commonly applied in experimental research. Moreover, the interpretation of ERG recordings from animals presents some difficulties since, in various types of ERG tests, the waves in animals significantly differ, unlike those in humans. It is important to note that numerous studies, especially on the development of new drugs, are based on animal models. For valid conclusions to be drawn from such studies, the ERG results from animals must be properly interpreted. Therefore, we collected information on the types of ERG tests used to diagnose DR in rodents and paid special attention to changes that indicated functional disorders in the retina, which probably precede vascular alteration [11]. The first part of this paper presents basic information on the types of ERG tests used in animal studies, and the second part describes the functional changes recorded in rodent retinas during the use of antioxidant treatment in DR, as well as details on the species and strains of the rodents and the ERG test type used in these studies. We hope that this report will be useful, especially in light of the latest reports in which DR was treated as a neuronal dysfunction rather than a vascular disorder.

## 2. Types of Electroretinography (ERG) Tests

ERG is an extracellular potential record arising from the currents that flow through the retina due to neuronal signalling. All retinal cells contribute to the ERG recorded at the cornea but have varying contributions to generating waves. Several factors affect the ERG record—primarily, the location of the cells in the retina, the stimulus conditions (the wavelength, temporal characteristics and energy) and the extent of the background illumination [12]. Moreover, an active extracellular electrode, which allows the ERG to be registered, can be placed on the cornea, in the vitreous or at different levels of the retina [13]. In addition, ERG parameters change throughout life; therefore, subjects of similar age should be compared [14,15].

**Figure 1:**
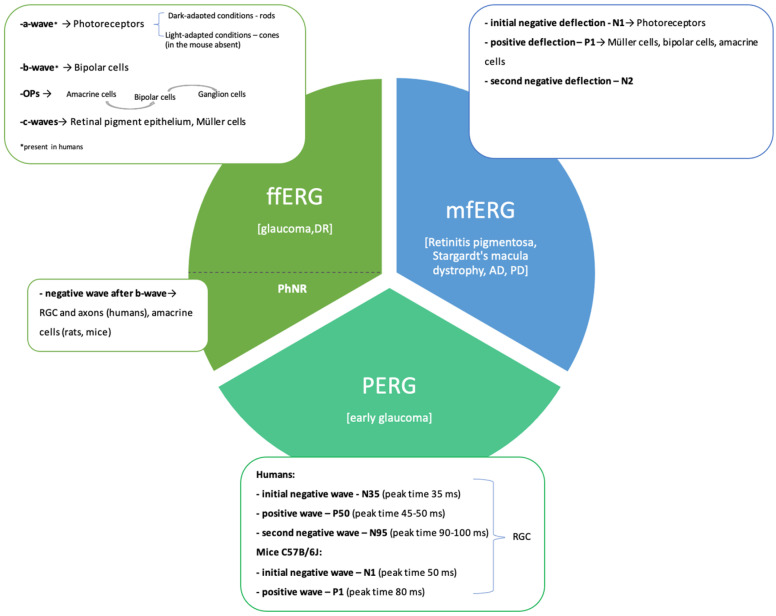
Types of waves recorded in various electroretinography tests. (ffERG—full-field ERG; mfERG—multifocal ERG; PERG—pattern ERG; PhNR—the photopic negative response; RGC—retinal ganglion cells; OPs—oscillatory potentials; AD—Alzheimer’s disease; PD—Parkinson’s disease).

There are three types of ERG: full-field ERG (ffERG), multifocal ERG (mfERG) and pattern ERG (PERG) (Figure 1) [16]. To standardise the results of ERGs performed in different laboratories, ISCEV has published guidelines, including different protocols, in a public-access website [10]. The ffERG test is often used for DR diagnosis and in studies on this ocular dysfunction. Therefore, it is described in detail in this section.

### 2.1. Full-Field ERG

FfERG is the most common clinical visual electrodiagnostic test that enables the evaluation of the retinal function under dark-adapted and light-adapted conditions [17]. In this method, the retina is illuminated evenly by a Ganzfeld stimulator that delivers diffuse flashes. The minimum period of dark adaptation (rod domination) should be 20 min, and light adaptation (cone domination), 10 min. The strengths of the flash stimulus used during standard dark-adapted state ERG are 0.01 cd × s/m^2^ (rod response), 3 cd × s/m^2^ and 10 cd × s/m^2^ (rod–cone response). Fast oscillations, known as ‘oscillatory potentials’ (OPs), are recorded in the 3 or 10 cd × s/m^2^ flask stimuli and may be noted as separate recordings. The light-adapted state has two parts: the response for a flash strength of 3 cd × s/m^2^, superimposed on a light-adapting background as single flashes (L, M- and S-cone responses) and at a frequency close to 30 Hz (L- and M-cone responses) [16]. The dark-adapted DR retina shows more functional pathologic changes than the light-adapted DR retina [18].

In ffERG, there are three main waves: the a-, b- and c-waves. The first negative wave (a-wave) appears immediately after the light stimulus is turned on and is followed by two positive waves (the b- and c-waves). The second positive c-wave is slower and has a lower amplitude than the b-wave. Finally, another positive wave, the d-wave, can be recorded at the termination of a light flash but is very rarely observed. Commonly, in human ERG, only a- and b-waves are visible. The a-wave reflects the response of the photoreceptors to light. The timing of the leading edge and the peak of dark-adapted a-waves evoked by strong stimuli can be used to estimate the rod sensitivity [12]. Under dark-adapted conditions, the rod photoreceptors dominate the a-wave, and under light-adapted conditions, the cone photoreceptors take over this role [12,19]. Most rodents are nocturnal; their retinas contain primary rod photoreceptors (97–99% of the total photoreceptor population), with few cones (mice, 2.9% and rats, 0.9%) [20,21,22,23]. As a result, in mouse ERG, the a-wave is absent at light-adapted levels [19].

The b-wave reflects the activity of the bipolar cells to light onset. In mice, it is dominated by rod or cone-depolarising bipolar cells depending on the adaptation state of the retina [19,24]. The cone ERG of mice, in contrast to that of primates and similar to that of rats, includes only a small contribution from hyperpolarising bipolar cell activity [25]. Moreover, on the ascending limb of the b-wave, both in humans and animals, OPs are observed. They include 4 or 5 low-amplitude, high-frequency waves, represent electrical currents generated by negative feedback pathways between amacrine cells, ganglion cells and bipolar cells, and are very sensitive to ischaemia. Therefore, OPs are good indicators of DR [1,26,27].

The c-wave is a composite of a positive wave produced by the retinal pigment epithelium (RPE) and a negative wave produced by Müller cells (called ‘slow PIII’) caused by the [K^+^]_0_ decrease [28]. Because the positive-going RPE contribution is larger than the negative-going Müller cell contribution, the c-wave is formed and, interestingly, is less positive in humans than in mice. Wu et al. indicated that the Kir4.1 channels presented in Müller cells of Kir4.1 mutant mice contributed to the K^+^ conductance [29].

The d-wave can be recorded only after applying long-duration (longer than 100 ms) light stimuli. Under such conditions, the ON and OFF phases of the ERG response are separate in time. A prolonged light stimulus enables normal functioning of the OFF pathway in a cone system and inhibits the ON pathway in both rod and cone systems. This wave depends entirely on AMPA/KA-type synaptic transmission, that is, between photoreceptors and OFF-centre bipolar cells [13].

**Figure 2:**
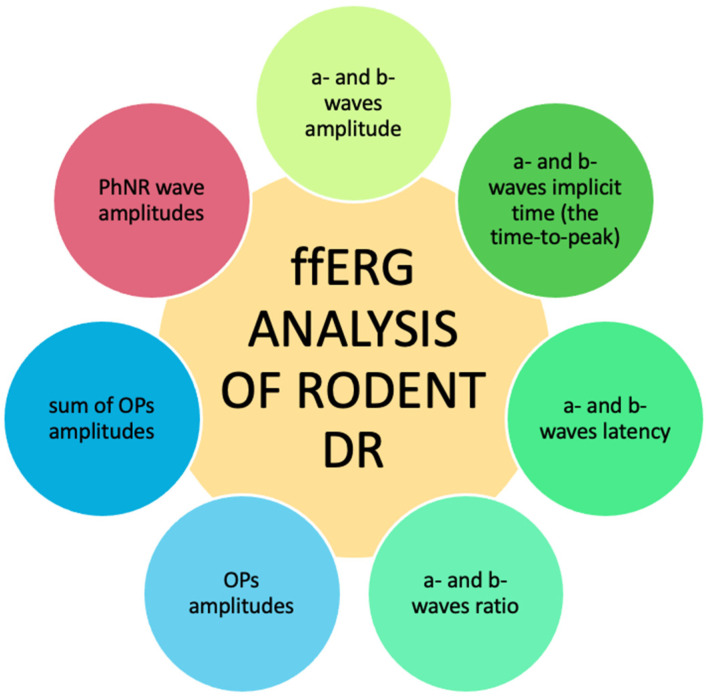
Results processing methods in rodent diabetes. (PhNR—the photopic negative response; OPs—oscillatory potentials).

ERG analysis of rodent DR can be based on measuring the a- and b-wave amplitudes or implicit times (the times-to-peak). Moreover, to compare the data obtained from different laboratories, the ratio of the b-wave to the a-wave is calculated. Such analysis enables the reduction in the differences caused by various recording conditions. Furthermore, the individual OP peak amplitudes of the dark-adapted 3.0 or the sum of the amplitudes of specified peaks are evaluated (Figure 2). The OP amplitudes are larger in mice than those recorded in primates or rats [30]. Interestingly, rats and mice do not have L-cones, and the spectral activity of their M-cones overlaps with that of their rods. Therefore, for rodent M-cone responses, scotopic stimulus calibrations are more appropriate than photopic calibrations [31]. Moreover, the recovery time of cone signals in mice and rats is longer than in primates [32,33]. This property should be taken into account when setting the parameters in the ERG test in rodents.

Additionally, ISCEV published the extended protocol for the ffERG test, called the photopic negative response (PhNR) of the flash ERG. PhNR is a negative-going wave that occurs after the b-wave in response to a brief flash [34]. This test is very useful for evaluating the condition of retinal ganglion cells (RGCs) and their axons. However, Miura et al. suggested that the PhNR in mice and rats, unlike in primates, arises from the activity of amacrine cells rather than of ganglion cells because the population of RGCs in rodents is smaller than in primates [35]. Reduction in PhNR amplitudes has been observed in different ocular diseases such as glaucoma [36,37,38], DR [39,40,41] and retinal artery occlusion [42,43]. Therefore, PhNR is often selected as an alternative test for retinal functional analysis in these diseases.

### 2.2. Multifocal ERG

MfERG is an electrophysiological test in which multiple areas of the retina are tested simultaneously using a contrast-reversing stimulus with an array of 64 or 103 black-and-white hexagons. This method enables the identification of damage to discrete retinal regions [44]. MfERG is traditionally recorded in photopic conditions and includes an initial negative deflection (N1: photoreceptors response), followed by a positive deflection (P1: Müller and bipolar cells and amacrine cells response) and a second negative deflection (N2) [45]. The test is often used to diagnose diseases with focal deficits, such as retinitis pigmentosa, Stargardt’s macular dystrophy and neurodegenerative diseases (e.g., Alzheimer’s disease and Parkinson’s disease).

### 2.3. Pattern ERG

PERG is an electrophysiologic test that provides information about the central retinal function in response to a pattern-reversing stimulus, a contrast-reversing checkerboard or a stripped stimulus [46]. PERG is a common method of assessing the macula and RGC functions and is altered early in glaucoma before histological RGC loss [47,48]. Moreover, this technique enables decision-making on whether a visual impairment is caused by retinal or optic nerve dysfunction. Standard transient PERG is recorded in response to low-contrast-reversal frequency stimuli (1–2 Hz), whereas steady-state PERG is evoked at higher temporal frequencies (above 5 Hz). Steady-state PERG is used to diagnose glaucoma [46,49,50]. The waveform in transient PERG consists of an initial negative wave (N35: peak time, 35 ms), followed by a positive wave (P50: peak time, 45 to 50 ms) and another negative wave (N95: peak time, 90 to 100 ms) [45]. In C57B/6J mice, the PERG waveform resembles that of humans and has the same components but different peak times (N1: peak time, 50 ms; P1: peak time, 80 ms; N2: peak time, 300 ms). Additionally, C57B/6J mice and DBA/2J mice are dissimilar because the P1 of DBA/2J mice is about 20 ms longer than that of C57B/6J mice [47].

In recent years, PERG has not been widely used in rodents, probably because it is considered technically difficult to record [49]. The essential condition for this test is proper fixation; the subject should be fixed at the centre of the screen. In humans, the fovea can be aligned using an indirect ophthalmoscope; however, this is more difficult to do in mice and causes some variability in the retinal stimulation. However, it is important to underline that the mouse retina has less difference in cell density between the central and peripheral regions than the primate retina [35].

Due to the development of PERG technology by using a single subcutaneous needle in the snout, the need to apply a corneal electrode has been eliminated, allowing for much more accurate measurements [48].

## 3. Effects of Selected Antioxidants on ERG in Diabetic Retinopathy

We collected data that showed the impact of selected antioxidants on the retinal function in rodent DR. Most of the antioxidants were selected based on the review of Garcia-Medina et al., a rich collection of currently studied antioxidants [51]. First, we carefully reassessed the substances mentioned in the review, with particular emphasis on the results of the ERG tests and more recent studies. Then, we identified other antioxidants whose impact on the retinal function in rodent DR was assessed via ERG. The most important information on the results of the presented studies is shown in Table 1.

### 3.1. Lutein, Docosahexanoic Acid and Ebselen

Lutein, a xanthophyll carotenoid, is an antioxidant that is not synthesised in vivo and thus needs to be acquired through the diet [70]. It is highly present in egg yolk and leafy green vegetables [71]. Taken regularly, it accumulates in the retina, particularly in the choroid and RPE cells, and has anti-inflammatory action that prevents choroidal neovascularisation [72]. It acts through several mechanisms, including scavenging free radicals, reducing the expression of inducible nitric oxide synthase and filtering blue light that may cause phototoxic damage to photoreceptor cells [73,74].

DHA is an omega-3 polyunsaturated acid that is highly present in the retina, brain and testis [75]. The retina acquires DHA from the plasma through a specific transporter, the major facilitator superfamily domain-containing protein 2 (Mfsd2a), the level of which, therefore, plays a crucial role in maintaining the required levels of this acid in the retina. The DHA in the outer segments of rodent retinas accounts for around 50% of all fatty acids [76]. Insufficient DHA intake is generally accepted as impairing the retinal function in experimental animals, including mice and rats. A DHA-rich diet has been shown to block the upregulation of inflammatory markers and prevent vascular pathologies in the retinas of diabetic rats [77].

Ebselen, a synthetic fat-soluble compound, can strengthen endogenous antioxidant defences in the retina [78]. It acts by activating nuclear factor erythroid 2-related factor 2 (Nrf2)-regulated genes. Notably, Nrf2 is found in Müller cells and glial cells in human and animal retinas [79]. Its expression in rats’ Müller cells has also been reported to be inhibited by high glucose levels, which induces oxidative stress [80].

Arnal et al. [52] assessed the effect of DHA and lutein on the function of the retinas of diabetic male Wistar rats. DHA and lutein were administered daily to the rats via oral gavage for 12 weeks, and 4 and 12 weeks after diabetes induction, scotopic ffERG was performed to measure the rats’ a- and b-waves. After 4 weeks, no change was observed between the groups. After 12 weeks, there was a significant decrease in the b-wave amplitude and an increase in latency in the diabetic group, unlike in the healthy rats. Treatment with lutein and DHA restored the b-wave amplitude and latency time to the control values. Wang et al. [53] observed similar results in Ins2Akita/+ heterozygote mice. Lutein was administered daily in the drinking water of the rats starting at 6 weeks of age. ERG was performed when the rats were 6.5 and 9 months of age. At both time points, the a- and b-wave amplitudes were decreased in the diabetic rats, unlike in the healthy rats, but the lutein treatment significantly ameliorated those responses at both time points. Miranda et al. [54] also assessed the role of lutein but administered with ebselen in albino male mice. The mice were orally given lutein and ebselen daily for 4 days after diabetes was induced. After 3 days of treatment, ERG was performed, and the results showed no difference between the diabetic, control and treated groups. Unfortunately, the period between the diabetes induction and the conduct of ERG was very short, and thus, the role of ebselen in preventing functional changes in the retinas in DR should be reassessed.

### 3.2. Melatonin and Superoxide Dismutase 3

Melatonin is a unique antioxidant that can easily cross all body barriers due to its lipophilic and hydrophilic characters [81]. Melatonin treatment has been reported to have a protective effect on superoxide dismutase (SOD) expression and to help maintain low levels of reactive oxygen species in rats’ retinas [82,83,84]. SODs are elements of endogenous antioxidant defences that protect tissues from oxidative damage [85]. Three types of SOD have been identified in mammalian tissues, including SOD1 (found in the cytosol and the nucleus), SOD2 (found in the mitochondria), and SOD3, also called ‘extracellular superoxide dismutase’ (EC-SOD). The latter is the only antioxidant enzyme-scavenging superoxide, specifically in the extracellular compartment. Diabetic patients have been noted to have higher concentrations of glycated SOD3 in serum, which may be related to a decrease in matrix-associated SOD3 [86].

Jiang et al. [55] analysed the effects of melatonin on functional changes in diabetic retinas. They administered melatonin to male Sprague Dawley rats via intraperitoneal injection for 12 weeks. The ffERG performed at the end of the treatment showed that in both the scotopic and photopic responses, the a- and b-wave amplitudes of the diabetic rats decreased significantly, unlike those of the healthy rats. Thus, the melatonin treatment strongly alleviated such responses. No difference in the a- and b-wave latencies between the diabetic and non-diabetic rats was reported. Salido et al. [56] performed a similar study using male Wistar rats in which they subcutaneously implanted a pellet of melatonin 48 h after streptozocin (STZ) injection and replaced the pellet every 15 days. Unfortunately, there are no data on the glycemia level that qualify rats as diabetics. After 12 weeks of treatment, scotopic ffERG was performed and showed that the melatonin prevented a decrease in the OPs and in the a- and b-wave amplitudes, which were significantly reduced in the diabetic rats compared to the healthy rats. No difference in the OPs and the a- and b-wave latencies between the groups was observed. These results have been replicated by other studies [57,58].

Lee et al. [40] analysed the impact of SOD3 on DR in male Sprague Dawley rats. SOD3 was injected intravitreally at the same time as STZ. FfERG was performed 1, 2, 4 and 8 weeks after diabetes induction. The scotopic responses showed reduced a-wave amplitudes in the diabetic rats compared to those with SOD3 at all time points, but some of the reductions were not statistically significant. Significant reductions in the a- and b-waves were observed only after 2 and 8 weeks. The photopic ffERG responses showed no statistically significant change in the a-wave amplitude but a significant reduction in the b-wave amplitude after 8 weeks. The PhNRs significantly decreased after 2 and 8 weeks. In the diabetic group, the PhNR amplitude significantly dropped in weeks 1 to 8, and the diabetic group with SOD3 treatment showed no such reduction until 4 and 8 weeks after diabetes induction. To sum up, SOD3 treatment attenuated the loss of the a- and b-wave amplitudes in the scotopic ffERG and of the b-wave amplitudes in the photopic ffERG of the diabetic rats.

### 3.3. Taurine

Taurine is an organic osmolyte that is widely spread in all ocular tissues [87,88,89,90]. Moreover, it is one of the most abundant amino acids in the retina and protects the retina from stress-related neuronal damage. It reaches higher concentrations in the intracellular than in the extracellular habitat due to the Na^+^-dependent specific transporter, which is upregulated by, for example, oxidative stress and low extracellular taurine levels and downregulated by, for example, high glucose concentrations [91]. Taurine is generally considered a poor scavenger of free oxygen radicals, but it scavenges hypochlorite, which may be crucial in the retina, where the hypochlorite-producing enzyme is located [92]. Taurine has been reported to attenuate an increase in the concentration of the vascular endothelial growth factor protein in diabetic rats [93] and to ameliorate biochemical retinal abnormalities caused by diabetes [92].

Fan et al. [59] assessed the role of taurine in preventing functional losses in the retinas of diabetic male Sprague Dawley rats through intraperitoneal injection or intragastric administration of taurine in drinking water daily for 4 weeks. Photopic ffERG was performed at 4 weeks after diabetes induction. The diabetic rats had significantly lower b-wave amplitudes than the healthy rats. The taurine supplementation improved the results, especially in the group treated with intraperitoneal injection.

### 3.4. Edaravone

Edaravone (3-methyl-1-phenyl-2-pyrazolin-5-one) is a free radical scavenger with proven anti-ischaemic effects. In 2001, it was accepted in Japan for the clinical management of acute ischaemic stroke [94,95,96]. It inhibits lipid peroxidation and vascular endothelium injury. It also showed a potential to slow down the progression of photoreceptor degeneration in light-induced retinal damage [97] and after retinal detachment [98].

Yuan et al. [60] assessed the impact of intraperitoneally injected edaravone on the retinal function in DR using male C57BL/6 mice. Edaravone was administered to the mice daily for 4 weeks after diabetes induction. At the end of the treatment, PERG was performed. Significant delays in the implicit times and reductions in the amplitudes of the P50 and N95 waves were observed in the diabetic mice. Although the edaravone treatment improved these results to a limited extent, it did not prevent significant negative changes, unlike in healthy rats. It would also be worth analysing how edaravone affects OPs, a- and b-wave amplitudes and implicit times.

### 3.5. Lisosan G

Lisosan G is an antioxidant obtained from the grains of *Triticum aestivum* [99]. Amato et al. [61] assessed its potential to prevent functional damage to the retinas of diabetic Wistar rats. It was administered starting a week after the diabetes onset, once daily via oral gavage for 4 weeks. Scotopic ffERG was performed once a week after diabetes induction. From the third week of diabetes, the a- and b-wave amplitudes decreased both in diabetic and treated rats, unlike in healthy rats. In the fourth week, the decreases in the a- and b-wave amplitudes were more marked in the diabetic group than in the treatment group, but in the fifth week, the outcomes in these two groups were similar. This may suggest that Lisosan G protects the inner and outer retinas from functional deficits. Moreover, Amamto et al. suggested in a recent experiment that this antioxidant may also reduce the pathological changes associated with glaucoma [100].

### 3.6. Hydrogen-Rich Saline

Hydrogen-rich saline (HRS) has recently gained much interest in various fields of medicine. Its exact mechanism of action remains unclear, but its main mechanisms are suspected to be alteration of gene expression, modulation of signal transduction and selective scavenging of OH radicals [101]. It has been reported to have neuroprotective properties in experimental cerebral ischaemia in rats and to reduce oxidative stress and inflammatory response in spinal cord injury in rats, thus increasing the efficiency of bone mesenchymal stem cell transplantation [102,103,104]. It has also been suggested to treat various cardiovascular diseases [105].

Feng et al. [62] analysed the effect of HRS on the retinal function in diabetic male Sprague Dawley rats. One week after diabetes induction, daily intraperitoneal administration of HRS was started. After 1 month of treatment, ffERG was performed. Significant reduction in the b-wave and OP amplitudes in the diabetic rats compared to the control group was reported but prevented by a daily HRS treatment.

### 3.7. Pigment Epithelium-Derived Factor

Pigment epithelium-derived factor (PEDF) in the eye is mainly expressed in the cornea, ciliary epithelium and retinal pigment epithelial cells and is secreted to the interphotoreceptor matrix [106,107]. It is known to have neuroprotective and antiangiogenic effects [108]. Its neuroprotective effect is based on its antioxidative abilities, and it has been shown to inhibit the degeneration of photoreceptors exposed to constant bright light [109].

Yoshida et al. [63] analysed the effect of PEDF on diabetic male Wistar rats. PEDF was administered intravenously three times a week for up to 4 weeks, after which ffERG was performed. The amplitudes of the a- and b-waves were decreased in the diabetic rats, unlike in the non-diabetic rats and the PEDF-treated diabetic rats, but the b-wave amplitude decreased more significantly.

### 3.8. Alpha-Lipoic Acid

Alpha-lipoic acid (ALA) is an antioxidant with strong anti-inflammatory effects [110]. Aside from direct free-radical-scavenging properties, it enables nuclear translocation of Nfr-2, thus increasing the expressions of SOD, catalase and glutathione peroxidase genes. Kim et al. [111] recently reported that ALA reduces oxidative stress and retinal injuries in diabetic mouse retinas by decreasing the interaction of AMP-activated protein kinase with O-linked β-N-acetylglucosamine. Johnsen-Soriano et al. [64] assessed the impact of ALA treatment on the retinal function in diabetic male albino mice. They intraperitoneally administered ALA daily for 3 weeks, after which they performed ffERG. The b-wave amplitudes significantly decreased in the diabetic mice compared to the other groups. Administration of lipoic acid restored the b-wave amplitudes to 77% of the control values.

### 3.9. Resveratrol

Resveratrol (3,5,4′-trans-trihydroxystilbene) is a polyphenolic phytoalexin with anti-inflammatory, neuroprotective and antioxidant properties [112]. It was reported to have a beneficial effect on various ophthalmic diseases, such as glaucoma [113], cataracts [114,115] and age-related macular degeneration (AMD) [116].

Zeng et al. [65] used diabetic Sprague Dawley rats to analyse the effect of resveratrol on the function of diabetic retinas. The treatment was started after the induction of diabetes and was administered via oral gavage once daily for 7 months. FfERG was performed after 1, 3, 5 and 7 months of treatment. The scotopic 0.01 ffERG showed no difference in the a- and b-wave implicit times (the time-to-peak) and the a-wave amplitudes between the healthy, diabetic and treated rats. The b-wave amplitude decreased in the diabetic rats after 3, 5 and 7 months with the protective effect of the treatment. The scotopic 3.0 ERG showed no difference in the implicit times of the a- and b-waves between the groups. The a- and b-wave amplitudes of the diabetic rats decreased at all time points. The treatment enabled recovery of the a- and b-wave amplitudes at all time points. As for the OPs, the implicit time of OP2 was delayed and its amplitude decreased in the diabetic rats at all time points, unlike in the healthy rats, but the resveratrol treatment ameliorated these results. The photopic ERG showed no significant change between the groups.

### 3.10. Açaí

Açaí fruits have recently attracted much interest from researchers in various fields of medicine. They are thought to have antioxidant and anti-inflammatory effects connected to the prevention of cardiovascular diseases and diabetes [117,118,119,120,121]. Their exact effects on various diseases remain unclear and are currently being investigated.

De Oliveira [66] analysed the effects of açaí supplementation on adult Swiss diabetic mice. After diabetes induction, a diet enriched with açaí was administered for 60 days. FfERG was performed after 30, 45 and 60 days of treatment. The combined scotopic and photopic responses showed decreased b-wave amplitudes in the diabetic mice, with the very strong protective effect of the açaí treatment maintaining the amplitudes at the level of those in the healthy mice at all time points. The photopic responses showed açaí’s protective effect after 30 and 45 days. On the 60th day, the amplitude of the açaí group was significantly lower than that of the control group but still higher than that of the diabetic mice.

### 3.11. Mixtures Containing Various Antioxidants

Canovai et al. [67] analysed the effects of a compound of cyanidin-3-glucoside, verbascoside and zinc—nutrients with antioxidant and anti-inflammatory properties—on the retinal function of Sprague Dawley rats. The compound was administered daily via oral gavage for 30 days after diabetes induction. After the treatment, scotopic ffERG was performed. The diabetic rats showed reduced a- and b-wave amplitudes, unlike the healthy rats. The rats treated with a low dose of the compound showed significantly higher b-wave amplitudes than the healthy rats, but their a-wave amplitudes differed only slightly. In the rats administered with a high dose of the compound, the a- and b-wave amplitudes were significantly higher than in the diabetic rats but still lower than in the healthy rats.

McClinton et al. [68] analysed the impact of carotenoid-rich carrot powder (that consisted of alpha-carotene, beta-carotene and carrot powder) on the retinal function of diabetic Wistar rats. The diet that contained carotenoid-rich carrot powder was administered for 3 weeks, after which diabetes was induced. Then, the diet was continued for 9 more weeks, at the end of which ffERG was performed. The diabetic rats showed decreased a- and b-wave amplitudes compared to the healthy rats. The healthy rats treated with carotenoid-rich carrot powder had higher amplitudes than the untreated rats. Interestingly, in the diabetic rats, the supplementation with carotenoid-rich powder had the opposite effect; the ERG showed further decreases in the amplitudes, unlike in the diabetic group. The amplitudes of the OPs in the diabetic group also decreased without the protective effect of the carotenoid-rich powder, but the frequency and onset of the OPs increased with the carotenoid-rich powder supplementation. The implicit times also decreased in diabetic rats without the protective effect of the carotenoid-rich powder supplementation. In the light-adapted ERG, the carotenoid-rich carrot powder supplementation was associated with increased cone-driven retina function in the control group but had the opposite effect in diabetic rats. The diabetes or the treatment did not affect the cone-driven OP amplitudes and the light-adapted b-wave implicit times.

Suryavanshi et al. [69] assessed the role of *Triphala churna* on the retinal function in rodent DR. *Triphala churna* is a compound of mixed fruits of *Terminalia chebula*, *Terminalia bellirica* and *Emblica officinalis* with antioxidant and anti-inflammatory potential [122,123]. Suryavanshi’s research teams reported that *Triphala churna* has a protective effect on diabetic nephropathy [124] and neuropathy [125]. This time, Suryavanshi et al. [69] induced diabetes in male Sprague Dawley rats and then divided them into three groups and administered 250, 500 and 1000 mg/kg of *Triphala churna* to them, respectively, via oral gavage once daily for 4 weeks. FfERG was performed at the end of the treatment. The diabetic rats showed significantly decreased a- and b-wave amplitudes compared to the healthy rats and the rats treated with 500 and 1000 mg/kg of *Triphala churna*. The latencies of the a- and b-waves significantly increased in the diabetic rats, and the 500 and 1000 mg/kg doses of *Triphala churna* showed protective effects.

## 4. Conclusions and Future Directions

The proper diagnosis of eye diseases and the assessment of disease progression are largely based on electrophysiology. Therefore, correct interpretation of ERG results is essential for evaluating functional changes in the visual pathway both in clinical and laboratory settings. Animal models, mainly rodent models, are commonly used in studies on the visual pathway, of which functional testing is a very important element. When analysing the effect of various drugs on the retina, we cannot focus only on the morphological or molecular changes and ignore the functional aspect, which provides the final confirmation of the effectiveness of a treatment. Thus, knowledge of the functional changes observed in specific ocular disorders in rats and mice and knowledge of differences between the retinal structures of humans and rodents due to, among others, the nocturnal lifestyle of rodents are pivotal. This study shows that among the tests for DR diagnosis in rodents, ffERG is accurate and the most commonly used, and PERG and PhNR tests are rarely used. Moreover, the dark-adapted DR retinas of the rodents showed more functional pathologic changes than the light-adapted DR retinas. Therefore, the results in this stage of ffERG should be considered in further studies. Additionally, researchers underline that the age, species and even strain or gender of animals have a huge impact on the functional recording, and this must be kept in mind when comparing ERG results between animals.

Furthermore, based on the studies we have reviewed, we can conclude that antioxidants generally protect the retina from functional losses. Their beneficial influence is expressed in the preserved amplitudes of the a- and b-waves and the OPs in diabetic animals supplemented with antioxidants. Notably, however, most of the studies we reviewed were conducted over a relatively short period of time. Moreover, in several of the studies, the prolongation of the drug exposure showed that the antioxidants were able to delay the onset of adverse changes but did not stop them. Future studies should concentrate on how long-term antioxidant supplementation affects the retinal function.

## Figures and Tables

**Table 1:** Effects of selected antioxidants on ERG in diabetic retinopathy (* after diabetes induction; ** after treatment induction; †—before diabetes induction; OA—oral application; IP—intraperitoneal injection; SIP—subcutaneously implanted pellet; IV—intravitreal injection; IVN—intravenously injection; ffERG—full-field ERG; PERG—pattern ERG; OPs—oscillatory potentials).

Antioxidant	Treatment		Administration Way/Frequency	Animal/Sex/Age	Diabetes Model	Level of Hyperglycemia	ERG				Ref.
	Start Point *	Duration Time					Test Type	Test Time Points **	Results		
									Diabetes Without Treatment	Diabetes with Treatment	
**LUTEIN**	3 days	12 weeks	OA (gavage)/daily	Wistar rats/male/no date	STZ	>200 mg/dL	Scotopic ERG	4 and 12 weeks	↓ b-wave amplitude (12 weeks); ↑ b-wave latency time (12 weeks)	b-wave amplitude and latency time restored to control values	[52]
	6 weeks of age	12 weeks	OA (drinking water)/daily	Ins2Akita/+ heterozygote mice/male/6 weeks	Akita	>250 mg/dL	Scotopic ERG	2 and 12 weeks	↓ a-and b-wave amplitudes (2 and 12 weeks)	↑ a-and b-wave amplitudes (2 and 12 weeks)	[53]
**LUTEIN + EBSELEN**	4 days	3 days	OA (gavage)//daily	Albino mice/male/no date	Alloxan	≥288 mg/dL	ffERG	3 days	no changes	no changes	[54]
**DHA (DOCOSAHEXAENOIC ACID)**	3 days	12 weeks	OA (gavage)//daily	Wistar rats/male/no date	STZ	>200 mg/dL	Scotopic ffERG	12 weeks	↓ b-wave amplitude; ↑ b-wave latency time	b-wave amplitude and latency time restored to control values	[52]
**MELATONIN**	3 days	12 weeks	IP/no date	Sprague Dawley rats/male/no date	STZ	≥300 mg/dL	ffERG	12 weeks	↓ a-and b-wave amplitudes	a- and b-wave amplitude restored to control values	[55]
	48 h or 3 weeks	12 weeks	SIP/every 15 days	Wistar rats/male/12 weeks	STZ	no data	Scotopic ffERG	12 weeks	↓ a-and b-wave amplitudes; ↓OPs	a- and b-wave amplitudes and OPS restored to control values	[56]
	24 h	8 weeks	IV/once a week	Sprague Dawley rats/male/no date	STZ	>300 mg/dL	ffERG	8 weeks	↓ a-and b-wave amplitudes	a- and b-wave amplitudes restored to control values	[57]
	1 week	12 weeks	OA (gavage)//daily	C57BL/6 mice/male/20 weeks	STZ	>250 mg/dL	ffERG	4, 8 and 12 weeks	↓ a-and b-wave amplitudes; ↑ implicit times	a- and b-wave amplitudes and implicit times restored to control values	[58]
**SOD3**	0 days (at the same time as STZ)	8 weeks	IV/3 injections (at the time of STZ injection and 1 and 2 weeks after STZ injection)	Sprague Dawley rats/male/8 weeks	STZ	>250 mg/dL	ffERG	1, 2, 4 and 8 weeks	↓ scotopic a- and b-waves amplitudes (2 and 8 weeks); ↓ photopic b-wave amplitude (8 weeks)	scotopic a- and b-wave amplitudes and photopic b-wave amplitude restored to control values	[40]
**TAURINE**	no data	4 weeks	IP and OA (gavage)//daily	Sprague Dawley rats/male/8 weeks	STZ	>250 mg/dL	Photopic ffERG	4 weeks	↓ b-wave amplitude	b-wave amplitude restored to control values (IP > OG)	[59]
**EDARAVONE**	0 days (at the same time as STZ)	4 weeks	IP/daily	C57BL/6 mice/male/6 weeks	STZ	>288 mg/dL	PERG	4 weeks	↑ implicit times; ↓ P50 and N95 wave amplitudes	P50 and N95 wave amplitudes restored to control values	[60]
**LISOSAN G**	1 week	4 weeks	OA (gavage)//daily	Wistar rats/no data/no date	STZ	>250 mg/dL	Scotopic ffERG	1, 2, 3, 4, 5 weeks	↓ a-and b-wave amplitudes (3 weeks)	a- and b-wave amplitudes restored to control values	[61]
**HYDROGEN-RICH SALINE**	1 week	4 weeks	IP/daily	Sprague Dawley rats/male/no date	STZ	>250 mg/dL	ffERG	4 weeks	↓ b-wave amplitude; ↓ OPs	b-wave amplitude and OPs restored to control values	[62]
**PEDF (PIGMENT EPITHELIUM-DERIVED FACTOR)**	48 h	4 weeks	IVN/3 times a week	Wistar rats/male/6 weeks	STZ	>250 mg/dL	ffERG	4 weeks	↓ a-and b-wave amplitudes	a- and b-wave amplitudes restored to control values (a-wave < b-wave)	[63]
**⍺** **-LIPOIC ACID**	4 days	3 weeks	IP/daily	Albino mice/male/no date	Alloxan	>288 mg/dL	ffERG	3 weeks	↓ b-wave amplitude	b-wave amplitude restored to control values	[64]
**RESVERATROL**	72 h	28 weeks	OA (gavage)//daily	Sprague Dawley rats/no date/15 weeks	STZ	>300 mg/dL	ffERG	1, 3, 5, 28 weeks	↓ Scotopic 0.01 b-wave amplitude (12 weeks); ↓ scotopic 3.0 a- and b-waves amplitudes (4 weeks); ↑implicit time; ↓ OP2 (4 weeks)	a- and b-waves amplitudes, OP2 amplitude and implicit time restored to control	[65]
**AÇAÍ**	48 h	9 weeks	OA/daily	Swiss mice/male and female/no date	Alloxan	>200 mg/dL	ffERG	4, 6, 9 weeks	↓ a-and b-wave amplitudes (4 weeks)	a- and b-waves amplitudes restored to control	[66]
**COMPOUND OF CYANIDIN-3-GLUCOSIDE, VERBASCOSIDE AND ZINC**	0 days (at the same time as STZ)	4 weeks	OA (gavage)//daily	Sprague Dawley rats/male/8 weeks	STZ	≥250 mg/dL	ffERG	4 weeks	↓ a-and b-wave amplitudes	a- (a high dose of compound) and b-waves (a low and high dose of compound) amplitudes restored to control	[67]
**CAROTENOID-RICH CARROT POWDER**	3 weeks **^†^**	12 weeks	OA (diet)/daily	Wistar rats/male/3 weeks	STZ	≥216 mg/dL	ffERG	12 weeks	↓ a-and b-wave amplitudes; ↓OPs amplitude; ↑ implicit times	↓ a-and b-wave amplitude; ↓OPs amplitude; ↓ a-and b-wave implicit times; ↓OPs implicit times	[68]
***TRIPHALA CHURNA***	4 weeks	4 weeks	OA/daily	Sprague Dawley rats/male/no date	STZ	>250 mg/dL	ffERG	4 weeks	↓ a-and b-wave amplitudes; ↑ a- and b-waves latencies	a- and b-waves amplitudes; ↓ a- and b-waves latencies	[69]
